# Autophagy Induced by Muscarinic Acetylcholine Receptor 1 Mediates Migration and Invasion Targeting Atg5 via AMPK/mTOR Pathway in Prostate Cancer

**DOI:** 10.1155/2022/6523195

**Published:** 2022-06-09

**Authors:** Qianhui Wang, Jinying Chen, Mi Zhang, Hong Wang, Yan Zeng, Yanping Huang, Chen Xu

**Affiliations:** Institute of Life Sciences, Chongqing Medical University, Chongqing 400016, China

## Abstract

Increasing numbers of researchers discovered the expression of muscarinic acetylcholine receptor 1 in human cancers, while its function in human prostate cancer is still unclear. Our present study focused on CHRM1 to clarify its role in mediating autophagy in prostate cancer. We used immunohistochemistry, western blotting, and immunofluorescence experiments to observe the expression of muscarinic acetylcholine receptor 1 both in nude mice with subcutaneous tumors and in prostate cancer cells. The autophagy was observed through transmission electron microscopy, western blotting, quantitative real-time PCR, and immunofluorescence. After that, we used lentivirus to establish CHRM1 and Atg5 knockdown models. Then, the migration and invasion abilities after knocking down muscarinic acetylcholine receptor 1 and Atg5 were detected by transwell assays. In addition, the AMPK/mTOR pathway-related targets were detected by western blotting. We found that muscarinic acetylcholine receptor 1 was abundantly expressed both in vitro and in vivo in prostate cancer. The overexpression of muscarinic acetylcholine receptor 1 positively regulated migration and invasion in tumor cells as well as the activation of autophagy. Muscarinic acetylcholine receptor 1 was highly correlated with Atg5 and activated the AMPK/mTOR signaling pathway. Downregulation of Atg5 inhibited cell autophagy in prostate cancer cells and the migration and invasion of prostate cancer cells. Meanwhile, abnormal expressions of AMPK/mTOR pathway-related proteins were found. In conclusion, the present findings indicated that muscarinic acetylcholine receptor 1 is highly expressed in prostate cancer cells and promotes cell invasion and migration of prostate cancer. Autophagy is activated in prostate cancer cells and the activation of muscarinic acetylcholine receptor 1 positively regulates autophagy in prostate cancer cells. Moreover, muscarinic acetylcholine receptor 1 induces autophagy-mediated cell migration and invasion by targeting Atg5 in prostate cancer cells via AMPK/mTOR pathway, which uncovered that regulating muscarinic acetylcholine receptor 1, identified in this study, can be a promising solution for treating prostate cancer.

## 1. Introduction

Prostate cancer (PCa) ranks second in cancer mortality in developed countries as a male malignant tumor [1]. Conventionally, castration therapy is recommended to be first chosen to treat advanced PCa [2]. However, after 10–24 months of the treatment process, most cancer patients will gradually become non-androgen-dependent, leading to a poor response to traditional castration therapy [3]. In such circumstances, more effective methods need to be discovered.

Autophagy is described as a process forming autophagy lysosomes during the process of fusing cytoplasmic proteins or organelles with lysosomes and degrading the contents it contains to meet the demand of self-renewal and metabolism in some organelles and cells [4–7]. Previous genetic studies on yeast unveil that several ATG genes, such as Atg5, play an important role in forming autophagosome [8]. Environmental changes within the cell allow PCa cells to respond adaptively to stress and survive [9]. As a critical element of the microenvironment, nervous system activation may also influence homeostasis, including the process of cell fate mentioned above. Meanwhile, it is guaranteed that autophagy is a complicated physiological process that is also highly environmentally dependent [10]. Accumulating studies focus on the link between them. Moreover, autophagy in prostate epithelial cancer provides survival for tumor cells in the face of physiological stress. It has a tight relationship with the resistance of PCa chemotherapy drugs and the sensitivity to radiotherapy [11,12]. Therefore, autophagy has recently been considered as the direction of new advanced PCa treatment.

Muscarinic acetylcholine receptor consists of seven transmembrane domains and is a G-protein-coupled receptor targeting the parasympathetic synaptic membrane [13]. Five subtypes exist in normal prostate, as well as prostate tumor issues, but the CHRM1, which is mainly expressed in glandular epithelial cells, is the major receptor subtype that activates Gq signaling [14–17]. It is reported in the literature that CHRM1 promotes the proliferation and metastasis of PCa [18–20]. However, the specific mechanism is still not uncovered.

Studies have demonstrated that the M1 receptor can regulate PI3K/AKT, MAPK/ERK, Wnt, Hedgehog, and other signaling pathways [21]. Also, it is shown that the signaling pathways resulting in autophagy include MAPK/ERK, AMPK [10,22–24], mTOR pathways, and Beclin1/PI3KIII complexes [25]. Previous studies have revealed that CHRM1 can promote tumor metastasis through the Hedgehog pathway [21]. However, no relevant data has been reported on the link between CHRM1 and autophagy, especially in terms of CHRM1 regulating changes in the tumor microenvironment through autophagy. Therefore, we suppose that the activation of autophagy affecting development in PCa may be related to CHRM1, which remains a mystery within the sphere of our knowledge.

In this work, we observed high expression of CHRM1 after subcutaneous tumor formation in nude mice and PCa cell lines and detected the autophagy induced by CHRM1 in PCa cell lines. Mechanically, we found that CHRM1 was positively correlated with Atg5 and inhibited the AMPK/mTOR signaling pathway. Our research suggested that CHRM1 can function as a significant component to mediate autophagy to regulate tumor development in PCa.

## 2. Materials and Methods

### 2.1. Cell Culture and Drugs

The cells of each cell line were derived from ATCC and stored in liquid nitrogen. After being passed 3 times, the cell lines were started to be used for experiments and the passage number was no more than 5. The mixed medium cell culture components were as follows: DMEM/F12 medium (Gibco, c11330500bt, Carlsbad, CA, USA), 10% fetal bovine serum (FBS, 04-001-1ACS, Biological Industries, Israel), and 1% antibiotic mixture (penicillin/streptomycin, Beyotime, c0222, Shanghai, China). RWPE‐1 cells were grown in keratinocyte serum-free medium (K‐SFM, Gibco, 17005042, Carlsbad, CA, USA) supplemented with 10% FBS and 1% antibiotic mixture. Cells were cultured in a humidified incubator (Esco, CLM-170B-8-NF, Singapore) containing 5% CO_2_ at 37°C. The starvation induction condition is serum withdrawal, and the remaining conditions are unchanged. The following drugs were used: carbachol (MedChemExpress, HY-B1208, Monmouth Junction, NJ, USA, dissolved in ddH_2_O), pirenzepine (HY-17037, dissolved in ddH_2_O), proteinase inhibitor E64 (HY-15282, dissolved in DMSO), pepstatin A (HY–P0018, dissolved in DMSO), bafilomycin A1 (HY-100558, dissolved in DMSO), and 3-methyladenine (HY-19312, dissolved in DMSO).

### 2.2. Subcutaneous Tumor-Bearing Experiments in Nude Mice

The 6-week-old male nude mice (Beijing HFK Bioscience, Beijing, China) were randomly divided into the treatment group and the control group, with 20 males in each group. PC-3 cells and LNCaP cells (1 × 10^7^ cells/mouse) were resuspended in saline and subcutaneously injected into the armpits of nude mice in the treatment group. The control group was injected with saline. Four weeks later, a tumor photograph was taken, and the mice were sacrificed by ether anaesthesia to bluntly exfoliate the tumor tissue. The tumor was fixed in 4% paraformaldehyde for 24 h and embedded in paraffin for immunohistochemical staining.

### 2.3. Immunohistochemical Staining

Immunohistochemistry (IHC) on previously described subcutaneous tumor-bearing experiments in nude mice was performed. The slides were dried at 60°C for 1 h, dewaxed, and then incubated in different concentration of alcohol for rehydration. Subsequently, sections were placed in sodium citrate buffer for 5 min at high heat and 20 min at low heat for antigen retrieval. After being blocked with goat serum for 30 min, the slides were incubated with antibody (CHRM1, ab180636, 1 : 2000, Abcam) at 4°C overnight according to the instructions of Histostain-SP kit reagent (sp0023, Beijing Biosynthesis Biotechnology, China). Then the reaction was visualized using Diaminobenzidine (ZSGB Biotechnology, China) and the slides were counterstained with hematoxylin. Stained sections were examined and photographed on a bright‐field microscope (BX53; Olympus) connected to cellSens standard imaging software (Olympus).

### 2.4. Cell Transfection Lentiviral Construction Stable Transgenic Strain

Cell transfection and lentiviral construction stable transgenic strain were performed following the instructions. Cells were laid in 12-well plates 1 day in advance and lentiviruses resuspended in DMEM/F12 were added dropwise on cells. Fresh complete culture medium was replaced with after 12 hours. Then, puromycin at a concentration of 5 *μ*g/mL was added for screening lentivirus‐infected cells. Transfection of a control shRNA at the same concentration served as a control. There was a knockdown CHRM1 virus solution, a knockdown Atg5 virus solution, and the corresponding control (GeneChem Co., Ltd., Shanghai, China). The target gene interference sequences were as follows (5′-3′): sh-NC: TTCTCCGAACGTGTCACGT; sh-CHRM1: GCACTCTGCAACAAAGCCTTC; and sh-Atg5: TTTCATTCAGAAGCTGTTT. The pEGFP-C1-LC3 plasmid was obtained from the Beijing Micro-Spin Gene Technology Company. The LC3 fluorescent double-labelled adenovirus Ad-mCherry-GFP-LC3B (Beyotime, Shanghai, China) was used.

### 2.5. Cell Migration and Invasion Test

In the migration experiment, fifty thousand cells (100 *μ*l, DMEM/F12) were uniformly added to the transwell upper chamber (8.0 *μ*m pore size, Corning, Vineland, NJ, USA), and the medium containing 20% serum was added to the lower chamber. The treatment conditions were added to the upper chamber, and the cells were cultured. Then, the cells were fixed with 4% paraformaldehyde and stained with a crystal violet dye solution (Beyotime, Shanghai, China). The cells were washed, the cells in the upper chamber were gently washed off with a cotton swab. Then, the cells were photographed under the microscope, counted, and analyzed. To perform the invasion test, a 1:5 dilution of Matrigel (356234, Corning, Vineland, NJ, USA) was placed in the chamber and was incubated in the incubator for 2 h. After the Matrigel coagulated, the upper chamber was inoculated with 100,000 cells in a 100 *μ*l volume with 5% serum; 20% serum medium was added to the lower chamber. The remaining steps are the same as those of the migration experiment.

### 2.6. Wound-Healing Assay

Cells were inoculated into six-well plates in the exponential growth period until they reached nearly 80% confluence. The cells were washed, different treatment conditions were added, and a straight line was drawn by a pipette tip. After 0/12/24 hours of incubation, the cells were gently rinsed and were photographed under the microscope.

### 2.7. RNA Extraction Reverse Transcription and qPCR

The treated cells were prepared in accordance with the RNA extraction kit (Bioer, Hangzhou, China). Follow the instructions to obtain RNA after adding lysate R1, lysate R2, washing solution, and eluent in sequence. RNA concentration was measured, then reverse-transcribed (TaKaRa, Kusatsu, Japan), and subjected to qPCR (SYBR: TaKaRa, Kusatsu, Japan). The samples were detected on a Real-Time PCR Detection (Bio-Rad Laboratories) under the following conditions: 95°C for 5 minutes, 40 cycles of denaturation for 3 seconds at 95°C, 30 seconds of annealing at 58°C, elongation at 72°C for 30 seconds, and extension at 65°C for 5 seconds. CFX Manager software (Bio-Rad CFX Manager 3.1) was used to analyze the data. Primer sequences are shown in Table 1.

### 2.8. Western Blot

Western blotting was performed via established methods [21]. RIPA (Beyotime, Shanghai, China) buffer mixed with protease inhibitor cocktail (Bimake, USA) was used to lyse collected cells. Supernatants of 12000 g were obtained after the lysates were centrifuged for 20 min . Protein concentrations were measured by BCA assay. The protein from each sample was subjected to SDS-PAGE before it was transferred to an activated polyvinylidene difluoride membrane and blocked with 5% BSA in TBST buffer containing 100 mM Tris-HCl (pH 7.5), 150 mM NaCl, and 0.1% Tween-20 for 2 h. Then, the membrane was incubated in the relevant antibody at 4°C overnight and subsequently incubated with the corresponding secondary antibody for 1.5 h at room temperature. Immunocomplexes were visualized with a ChemiDoc Imaging machine (Bio‐Rad, CA, USA).

Antibodies against the following compounds were used: LC3 (ab192890, 1 : 2000, Abcam), CHRM1 (ab180636, 1 : 2000, Abcam), p-AMPK (#2535, 1 : 1000, CST), mTOR (#2983, 1 : 1000, CST), p-mTOR (#5536, 1 : 1000, CST), P62 (#8025, 1 : 1000, CST), Beclin 1 (#3495, 1 : 1000, CST), Atg5 (#12994, 1 : 1000, CST), Atg7 (#8558, 1 : 1000, CST), Atg12 (#4180, 1 : 1000, CST), Atg16 L (#8089, 1 : 1000, CST), E-cadherin (#3195, 1 : 1000, CST), N-cadherin (#13116, 1 : 1000, CST), Vimentin (#5741, 1 : 1000, CST), and GAPDH (#97166, 1 : 5000, CST). The secondary antibody was from Bioss (bs-0295G, bs-0368G, 1 : 5000).

### 2.9. Immunofluorescence Experiment

4% of paraformaldehyde was used to fix the cells. After blocking, the cells were probed with antibody (CHRM1, ab180636, 1 : 500, Abcam; LC3: ab192890, 1 : 500, Abcam)) followed by incubation with FITC or Cy3-conjugated secondary antibody (Bioss, bs-0295G-FITC,1/200 dilution; Proteintech, SA00009-2, 1/200 dilution). Then, DAPI was used to stain the nuclei by incubating cells, and a confocal microscope (NIKON, A1R) was made use of visualizing these cells.

### 2.10. Transmission Electron Microscopy

After the cells were attached, the cells were diluted with 0.1% trypsin and centrifuged at 800 rpm for 5 min. All except 1.5 ml of supernatant was discarded, and the 1.5 ml of liquid was transferred to the EP tube and centrifuged at 1200 rpm for 10 min. After the supernatant was absorbed, a special fixative was slowly added to the tube wall for electron microscopy without damaging the cell mass. Thin slices were cut with an ultramicrotome (EM UC7/UCT, Leica, Germany) and stained with 1% uranyl acetate and Reynold's lead citrate and observed under an electron microscope (JEM 1400-plus, JEOL, Japan).

### 2.11. Data Statistics

All data are expressed as the mean ± standard deviation from at least three independent experiments, as indicated with the significance score (^*∗*^*P* < 0.05; ^∗∗^*P* < 0.01; ^*∗∗∗*^*P* < 0.001; and ^*∗∗∗∗*^*P* < 0.0001) in the figure legends. Experimental data were statistically analyzed using GraphPad software (GraphPad Prism 6.01, USA).

## 3. Results

### 3.1. CHRM1 Is Highly Expressed in PCa

**Figure 1:**
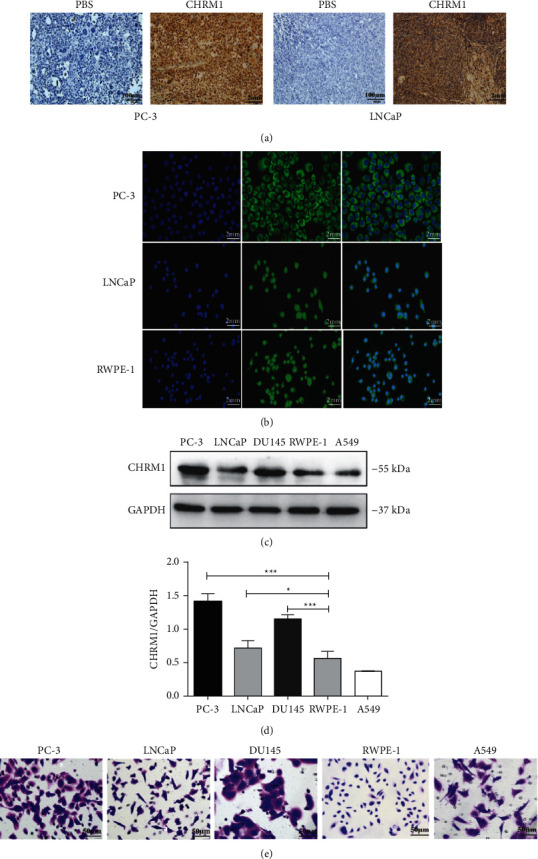
CHRM1 is highly expressed in PCa. (a) Immunohistochemical staining of CHRM1 in subcutaneous tumor-forming tumors of nude mice. Scale bar: PBS: 100 *μ*m. CHRM1: 2 mm. (b) Immunofluorescence detection of CHRM1 in PC-3, LNCaP, and RWPE-1 cells in which blue fluorescence is DAPI and green fluorescence is CHRM1. (c) Western blotting was performed to test CHRM1 expression in the human PCa cell lines PC-3, LNCaP, and DU145, the normal human prostate cell line RWPE-1, and the lung cancer cell line A549. (d) Quantification of the rate of CHRM1/GAPDH in cells. Data represent the mean ± SD of three experiments. ^*∗*^*P* < 0.05; ^∗∗∗^*P* < 0.001. (e) Cell crystal violet staining of PC-3, LNCaP, DU145, RWPE-1, and A549.

To determine whether CHRM1 plays a part in both PCa proliferation and tumor formation, we first used immunohistochemistry after subcutaneous tumor formation in nude mice to explore the expression of CHRM1. Immunohistochemical results showed that CHRM1 was abundantly expressed in tumor tissues of nude mice bearing PCa cells (Figure 1(a)). Then, immunofluorescence experiments showed that CHRM1 was expressed in the whole cell and mainly localized in the cell membranes and cytoplasm in human PCa cell lines PC-3 and LNCaP and the normal human prostate cell line RWPE-1 (Figure 1(b)). We compared the levels of CHRM1 expression in PC-3, LNCaP, human PCa cell line DU145, RWPE-1, and the lung cancer cell line A549 via WB analysis. The protein expression level of CHRM1 was much higher in the three PCa cell lines (Figures 1(c) and 1(d)). Cell crystal violet staining was used to observe each cell line (Figure 1(e)). These results suggest that CHRM1 is highly expressed in PCa cell lines.

### 3.2. Autophagy Is Activated in PCa Cells

**Figure 2:**
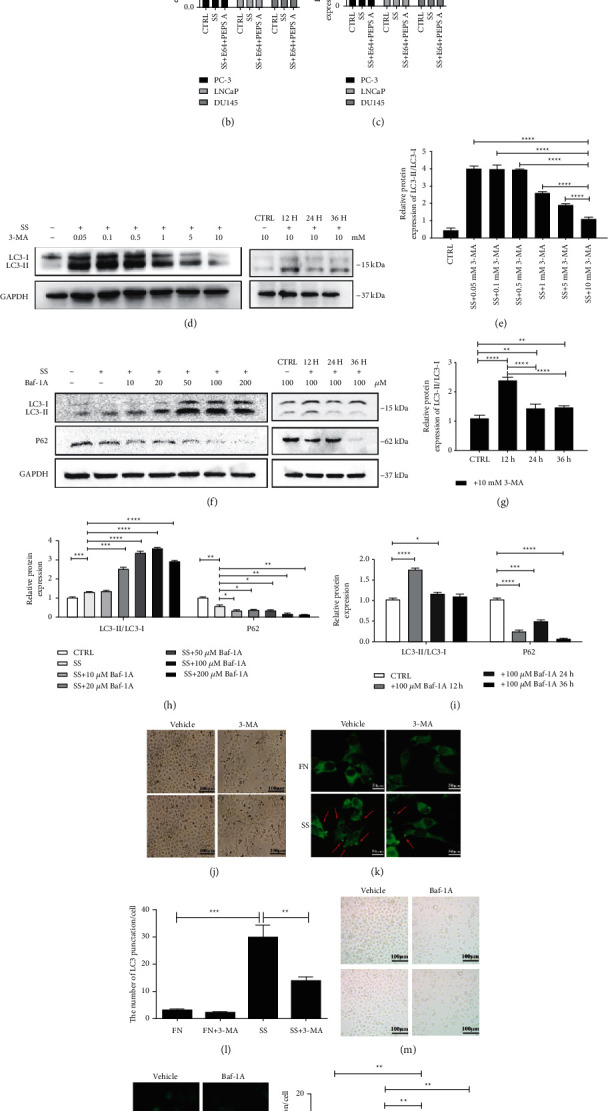
Autophagy is activated in PCa cells. (a) Western blotting was used to detect the reduction of P62 and the ratio of LC3-II/LC3-I by serum starvation (SS) in PCa cells for 24 h with or without E64 and PEPS A. (b-c) Relative quantification of the protein expression of P62 and LC3-II/LC3-I in Figure 2(a). (d, f) Western blot results selected the best time and concentration of autophagy inhibition. (e, g, h, i) Relative quantification of the protein expression of LC3-II/LC3-I and P62 in Figures 2(d) and 2(f). (j, m) Cell morphology under a light microscope in FN or SS conditions after 10 mM 3-MA for 24 h or 100 *μ*M Baf-A1 for 12 h. (k, n) PC-3 cells were transfected with pEGFP-C1-LC3 plasmid and cultured in FN or SS conditions and treated with 10 mM 3-MA for 24 h or 100 *μ*M Baf-A1 for 12 h. A fluorescence microscope was used to film cells to determine the expression of LC3-II. Red arrows pointed to green puncta. (l, o) Quantification of the numbers of LC3 puncta/cell in Figures 2(k) and 2(n). ^*∗*^*P* < 0.05; ^∗∗^P < 0.01; ^*∗∗∗*^*P* < 0.001; and ^*∗∗∗∗*^*P* < 0.0001.

To detect autophagy in various PCa cell lines, we detected the reduction of p62 and the conversion from LC3-I to LC3-II by serum starvation (SS) via western blotting, which respectively accounted for the activation of autophagy as biological markers. After hunger-induced autophagy, autophagy levels were increased in three PCa cell lines. With the use of lysosomal protease inhibitors, namely, E64 and Pepstatin A, the expression levels of p62 continued to be reductive; meanwhile, the ratio of LC3-II to LC3-I increased more (Figures 2(a)–2(c)). Western blotting was performed to determine the best time and concentration of autophagy inhibition after treating cells with autophagy inhibitors, respectively, which were 24 h and 10 mM for 3-methyladenine (3-MA) and 12 h and 100 *μ*M for bafilomycin A1 (Figures 2(d)–2(i)). Cell morphology was shown under light microscopy under full nutritional (FN) conditions or SS conditions, and the cells were slightly curled after the addition of 10 mM 3-MA for a day or 100 *μ*M Baf-1A for 12 h (Figures 2(j) and 2(m)). In quest of the localization and expression of autophagy in PCa cells, the pEGFP-C1-LC3 plasmid was transfected into PC-3 cells, with or without 3-MA or Baf-1A under FN and SS conditions. A large number of green puncta clustered in the cytoplasm under starvation conditions and the puncta decreased significantly after the addition of 3-MA or Baf-1A, suggesting that autophagy was inhibited (Figures 2(k), 2, 2(n), and 2(o)). Above all, autophagy is activated in PC-3, LNCaP, and DU145 under starvation condition. Therefore, we selected these three prostate cancer cell lines for the next test related to autophagy.

### 3.3. The Activation of CHRM1 Positively Regulates Autophagy in Prostate Cancer Cells

**Figure 3:**
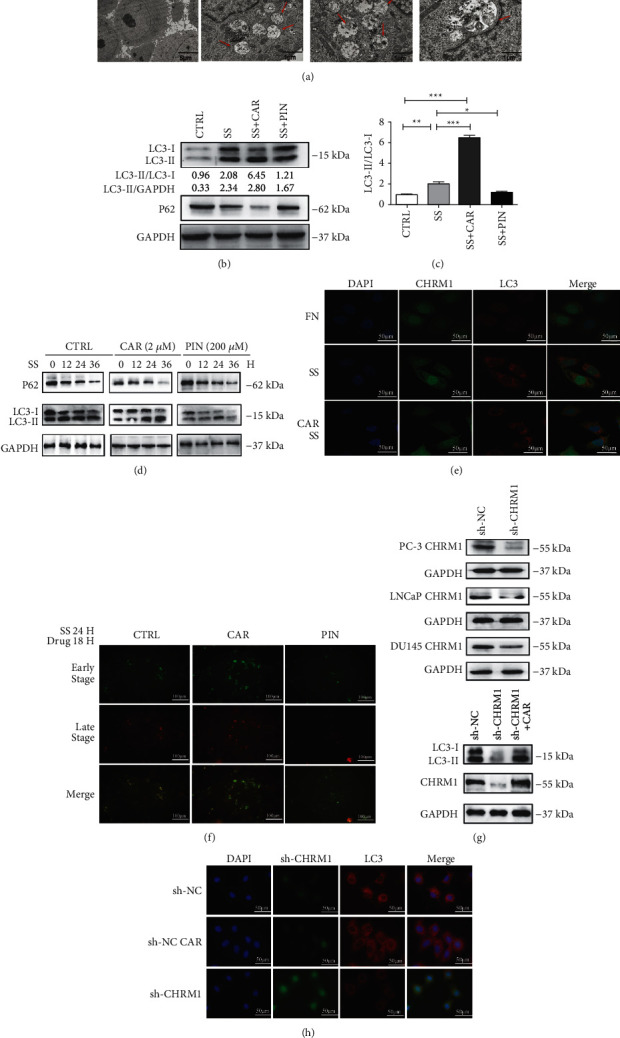
The activation of CHRM1 positively regulates autophagy in prostate cancer cells. (a) TEM for PCa cell after adding different concentrations of CAR. Red arrows pointed to autophagosomes with materials to be digested (CTRL: 4000X; CAR:15000X). (b) Western blotting of protein expression of p62, LC3-II or GAPDH treated with 2 *μ*M carbachol or 200 *μ*M pirenzepine for up to 24 h in the condition of serum-free medium or complete medium (CTRL) for up to 36 h. (c) Statistics of LC3-II/LC3-I ratio were obtained from Western blot results in Figure 3(b). Data in C are means ± SD. ^*∗*^*P* < 0.05; ^*∗∗*^*P* < 0.01; and ^∗∗∗^P < 0.001. (d) Western blotting of protein expression of p62, LC3-II, or GAPDH incubated in medium lacking serum for 36 h after the addition of carbachol or pirenzepine compared to nonspecific drugs in PC-3 cells. (e) LC3 puncta were analyzed by immunofluorescence after treatment with 2 *μ*M carbachol for 24 h under starvation conditions. Nuclei were stained with DAPI. Scale bar: 10 *μ*m. (f) PC-3 cells were transfected with Ad-mCherry-GFP-LC3B and cultured without serum for 24 h and treated with pirenzepine (200 *μ*M) and carbachol (2 *μ*M) for 18 h. A fluorescence microscope was used to determine the direction of autophagy flow by the expression of LC3-II. (g) Western blotting of the expression of CHRM1 after knocking down CHRM1 in PCa cell and the expression of LC3 in PC-3-sh-CHRM1 cell after adding CAR. (h) LC3 puncta were analyzed by immunofluorescence after knocking down CHRM1 in PC-3 for 24 h. DAPI was used to stain nuclei. sh-CHRM1: green fluorescence. Scale bar: 10 *μ*m.

To investigate the relevance between CHRM1 and autophagy in PCa cell lines, we photographed cells with transmission electron microscopy, a common tool of ultrastructural pathology. Then, we found that numerous autophagic structures appeared in the cytoplasm after the addition of the nonspecific muscarinic receptor agonist carbachol, suggesting that autophagy was activated (Figure 3(a)). Then, accumulation of the autophagy marker LC3 and degradation of p62 were detected after adding the CHRM1-specific antagonist pirenzepine and agonist CAR to PC-3 cells after starvation induction. It was found that CAR could activate the autophagy level by WB analysis, while PIN could inhibit its level in a statistically significant manner (Figures 3(b) and 3(c)). In PC-3 cells, after CAR was added to cultures in medium lacking serum for 36 h, p62 decreased, and LC3-II further increased. In addition, the autophagy levels in the CAR-treated cells increased with time compared to the control group. However, when PIN was added, the result was the opposite (Figure 3(d)). Similarly, the microscopic confocal results from the immunofluorescence experiment showed that after the addition of CAR in the starvation condition, LC3 red particles were significantly more than those in the starvation group and nutrition group, further suggesting enhanced autophagy (Figure 3(e)). Next, we transfected PC-3 cells with Ad-mCherry-GFP-LC3B and determined the direction of autophagy flow by comparing the fluorescence diagram results of CAR and PIN added to the control group after starvation-induced autophagy. Red fluorescence appeared to be higher after CAR treatment, and the most yellow spots in the composite figure showed a significant difference (Figure 3(f)). In order to further study the relationship between CHRM1 and autophagy, we used lentivirus to infect PC-3, LNCaP, and DU145 cells to establish cell lines with stable CHRM1 knockdown for subsequent mechanisms. It was found that the protein expression levels of LC3-II were inhibited after knocking down CHRM1. After adding CAR, the expression of LC3-II in PC-3-sh-CHRM1 cell had a certain response (Figure 3(g)). When CHRM1 was knocked down in PC-3 cell, the fluorescence intensity of LC3 declined compared with the control group (Figure 3(h)). It follows that the activation of CHRM1 positively regulates autophagy in prostate cancer cells.

### 3.4. CHRM1 Mediates Autophagy to Regulate the Migration and Invasion in PCa

**Figure 4:**
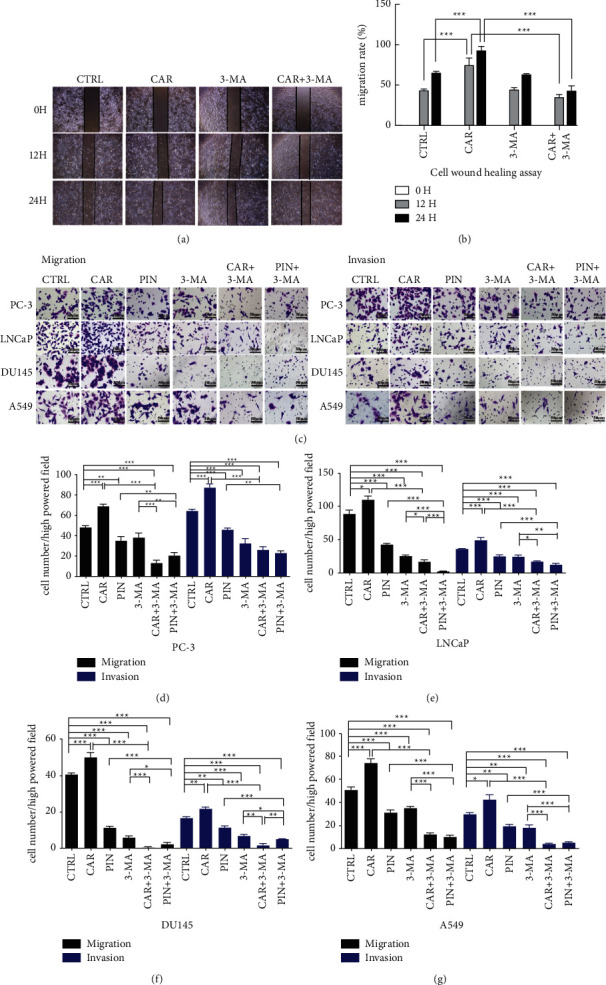
CHRM1 mediates autophagy to regulate the migration and invasion in PCa. (a) Wound-healing assay was established in 4 groups to detect PC-3 migration ability in three different periods (0, 12, and 24 h). The images were taken at a magnification of 40x. (b) Data from wound-healing assay using ImageJ for statistics, migration rate% = (Area 0 HR − Area XHR)/Area 0 h%. Each sample was run in triplicate. (c) Images of cell migration and invasion assays of the four tumor cell lines upon CAR, PIN, 3-MA, 3-MA + CAR, or 3-MA + PIN administration. The images were taken at a magnification of 400x. Scale bar: 1 mm. (d-g) Quantification of migrated (invaded) cell numbers in the cell migration (invasion) assays. Cells were counted in five random fields per section. ^*∗*^*P* < 0.05; *∗∗P* < 0.01; and ^∗∗∗^*P* < 0.001.

According to reports, CHRM1 overactivation can positively regulate cancer metastasis [21], and CHRM1 activation may enhance cell autophagy, so we speculated whether CHRM1-induced cell autophagy was involved in regulating prostate cancer. Next, 2 *μ*M carbachol and 200 *μ*M pirenzepine for 24 h and the autophagy inhibitor 3-MA were selected for the following tests [21]. The wound-healing assay tested the migration ability of PC-3 cells. We set up 4 groups at different times and found that adding 3-MA in the presence of CAR could reduce CAR-to-cell migration levels, which was statistically significant (Figures 4(a) and 4(b)). We used cell migration and invasion assays to detect relevant abilities of CHRM1 in order to find the relationship between CHRM1 and induced autophagy. Several treatment groups were set to determine the effect of CHRM1 on the migration and invasion of PCa cells and A549 cell. It was found that CAR could enhance the migration and invasion abilities, PIN led to opposite results, and adding 3-MA also inhibited cell migration and invasion. Based on the addition of agonists or inhibitors, autophagy was blocked, and the cellular migration and invasion abilities were more inhibited in a statistically significant manner (Figures 4(c)–4(g)). It is suggested that CHRM1 may enhance tumor cell migration and invasion abilities by activating the autophagy levels.

### 3.5. CHRM1 Induces Autophagy-Mediated Cell Migration and Invasion by Targeting Atg5 in Prostate Cancer Cells

**Figure 5:**
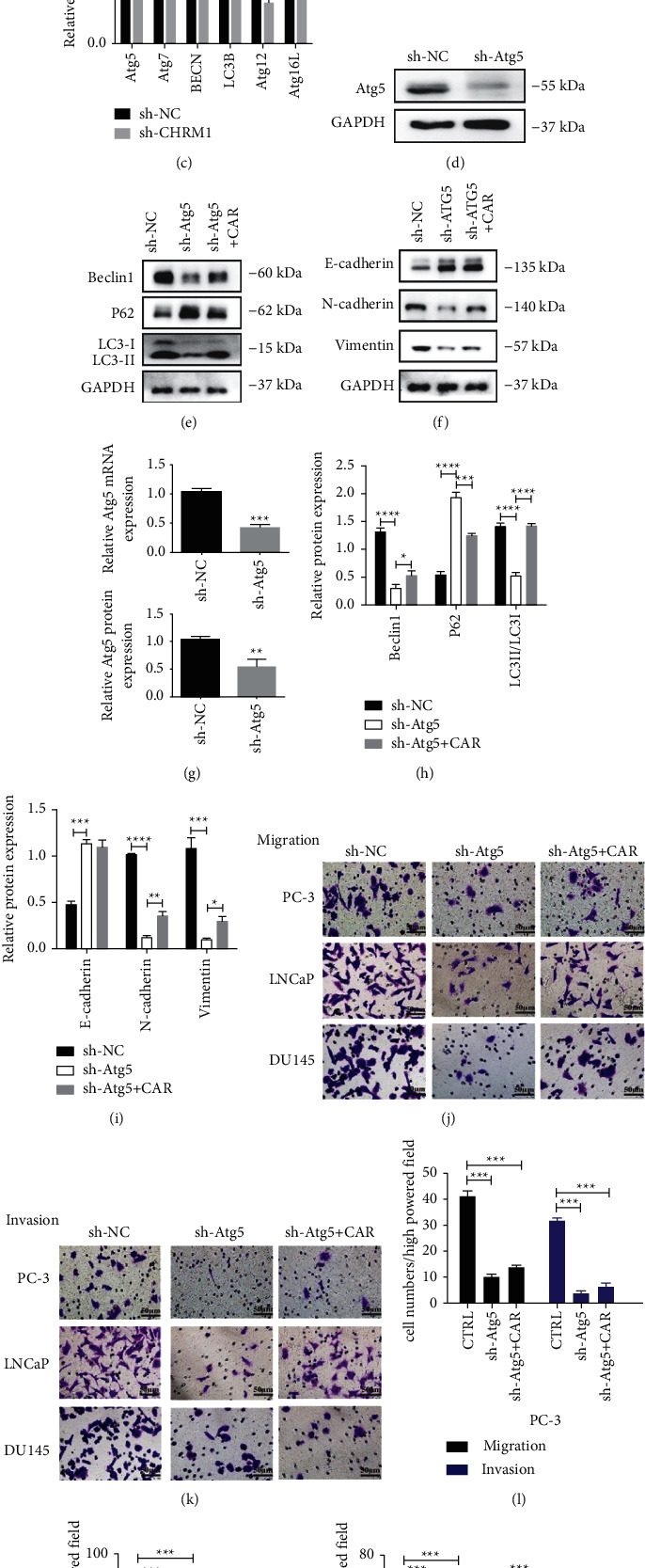
CHRM1 induces autophagy-mediated migration and invasion by targeting Atg5 in prostate cancer cells. (a–c) Determination of the autophagy-associated genes by western blotting and qPCR. ^*∗*^*P* < 0.05; ^∗∗^*P* < 0.01; and ^∗∗∗∗^*P* < 0.0001. (d, g) Determination of efficiency of knocking out of Atg5 by Western blotting and qPCR. Data in western blot and qPCR are means ± SD (*n* = 3); ^*∗∗*^*P* < 0.01; ^*∗∗∗*^*P* < 0.001. (e, h) Determination and quantification of autophagy-related proteins in PC‐3-sh-NC and sh-Atg5 cells treated with CAR by western blotting. ^*∗*^*P* < 0.05; ^*∗∗∗*^*P* < 0.001; and ^∗∗∗∗^*P* < 0.0001. (f, i) Determination and quantification of the expressions of E-cadherin, N-cadherin, and Vimentin in PC‐3 sh-NC and sh-Atg5 cells treated with CAR by western blotting. ^*∗*^*P* < 0.05; ^*∗∗*^*P* < 0.01; ^*∗∗∗*^*P* < 0.001; and ^∗∗∗∗^*P* < 0.0001. (j, k) Images of migration and invasion of the three tumor cell lines upon sh-NC, sh-Atg5, or sh-Atg5+CAR administration. Scale bar: 1 mm. (l–n) Quantification of migrated (invade) cell numbers in the cell migration (invasion) assays. Cells were counted in five random fields per section. ^*∗∗*^*P* < 0.01.

To further illustrate how CHRM1 regulates autophagy-mediated cell migration and invasion, the mRNA levels and protein expression levels of Atg5, Atg7, Atg12, Atg16 L, and BECN1 (Beclin1), known as highly associated with autophagy, were detected in PC‐3 cell transduced with a shRNA targeting CHRM1 (sh-CHRM1). We found that the expression of each was inhibited after knocking down CHRM1 (Figures 5(a)–5(c)), suggesting that the upregulation of CHRM1 correlates with the accumulation of the autophagy-related genes, especially Atg5. Knockdown of atg5 (Figures 5(d) and 5(g)) suppressed the expression levels of Beclin1; however, it promoted the expression of P62. After adding CAR, the expressions of Beclin1 and the ratio of LC3II to LC3I in PC-3 sh-Atg5 cells were elevated, and the expression of P62 decreased (Figures 5(e) and 5(h)). Then, we determined the protein expressions of E-cadherin, N-cadherin, and Vimentin in PC-3 sh-Atg5 cells and we found that downregulating Atg5 elevated the level of E-cadherin and suppressed the expressions of N-cadherin and Vimentin (Figures 5(f) and 5(i)). We next performed transwell assay in a sh-NC cell, sh-Atg5 cell, and sh-Atg5 cell treated with CAR in three tumor cells and observed a significant decrease (Figures 5(j)–5(n)), suggesting that knockdown of Atg5 weakened the migration and invasion abilities in PCa cells. Our study revealed that CHRM1 induces autophagy-mediated cell migration and invasion by targeting Atg5 in prostate cancer cells.

### 3.6. CHRM1 Induces Autophagy via AMPK/mTOR Pathway in Prostate Cancer Cells

**Figure 6:**
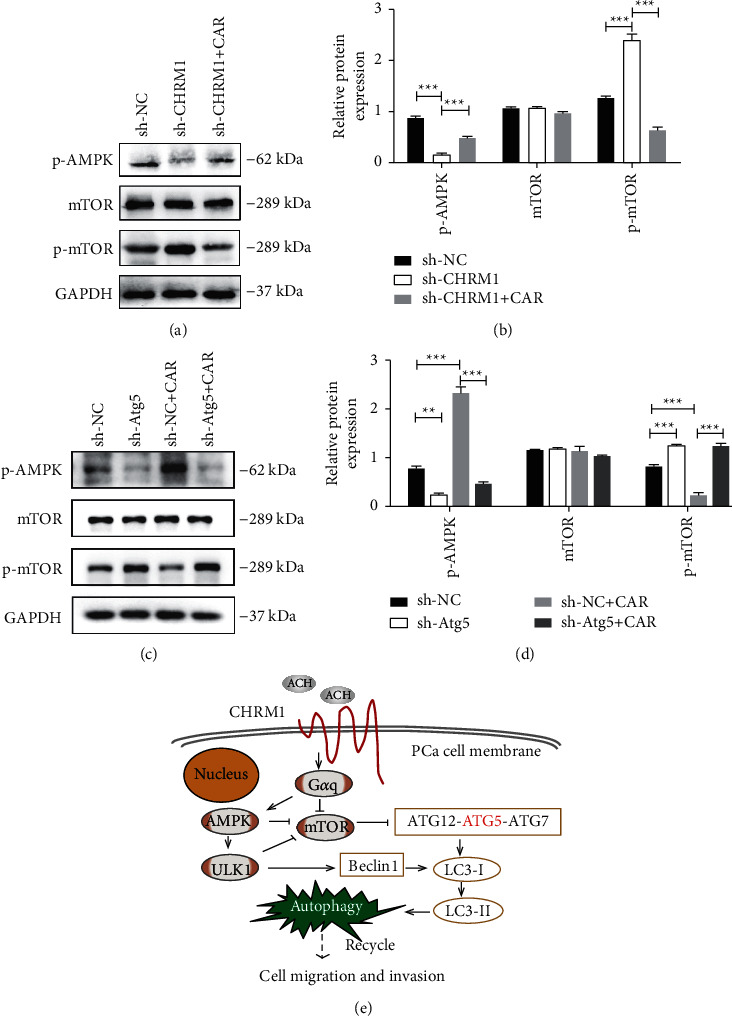
CHRM1 induces autophagy via AMPK/mTOR pathway in prostate cancer cells. (a-b) Determination and quantification of pathway-related protein expression levels in PC-3 sh-NC and sh-CHRM1 cells by western blotting. ^*∗∗∗*^*P* < 0.001. (c-d) Determination and quantification of changes of pathway-related protein in PC‐3 sh-NC and sh-Atg5 cells by western blotting. ^∗∗^*P* < 0.01; ^*∗∗∗*^*P* < 0.001. (e) Graphical schematic of the autophagy induced by CHRM1, which mediates migration and invasion targeting Atg5 via AMPK/mTOR pathway in prostate cancer.

Since AMPK/mTOR signaling pathway is involved in basic functions of cells, such as migration and proliferation; based on the former conclusions and studies [3], we reasonably speculated that CHRM1 may play a role in AMPK/mTOR signaling to regulate cell autophagy. Then, we knocked down CHRM1 in PC-3 cells and found that phosphorylated AMPK protein expression level decreased. In contrast, phosphorylated mTOR level increased (Figures 6(a) and 5(b)), suggesting that regulation of CHRM1 affects AMPK/mTOR pathway. Furthermore, we detected the levels of the pathway-related protein by western blotting, and we observed marked upregulation of p-mTOR and downregulation of p-AMPK in PC-3 sh-Atg5 cells. Intriguingly, compared with PC-3 sh-NC cells, PC-3 sh-Atg5 cells failed to restore the expression of p-AMPK and p-mTOR even in the presence of CAR, indicating that CHRM1 activates AMPK/mTOR pathway by targeting Atg5 in the prostate cancer cells (Figures 6(c) and 6(d)). The autophagy in PCa induced by CHRM1 was summarized with a model, which activates the AMPK/mTOR pathway by linking the Gq protein, acts on Atg5 and other autophagy-related genes, and induces autophagy, thereby enhancing the migration and invasion ability of tumor cells (Figure 6(e)).

## 4. Discussion

Muscarinic acetylcholine M1 receptor, the mainly expressed receptor subtype in the parasympathetic nervous system, has been shown to regulate cancer progression in a number of tumors, especially breast and pancreatic cancers [26–28]. It has been proved that the regulation of CHRM1 in prostate cancer involves a variety of programmed cell death processes. The stimulation of CHRM1 in prostate cancer can elevate the abilities of cell invasion and metastasis [28]. However, even if it has been reported that CHRM1 could be positively correlated with PCa progression, the possible mechanism remains uncharacterized. High expression of CHRM1 in PCa was observed by evaluating it in nude mice with subcutaneous tumors and in PCa cell lines. By adding cholinergic receptor agonist named carbachol (CAR) and its specific antagonist named pirenzepine, CHRM1 was detected to be positively subject to autophagy-mediated cell migration and invasion. Subsequently, this conclusion was proved in the knockdown cell model of CHRM1. Above all, CHRM1 modulated cell autophagy by targeting Atg5 via AMPK/mTOR signaling pathway because knocking down Atg5 distinctly abolished the effect of CAR on PCa cells and changed the expression of the AMPK/mTOR signaling pathway-related proteins.

Plenty of scientific studies support the contact between the neuroendocrine system and the tumor microenvironment [29–31]. Recent evidence suggests that muscarinic acetylcholine receptors play an indispensable part in assisting the parasympathetic nervous system with the development and progression of PCa [21,32,33]. Indeed, our research verified that CHRM1, the most expressed subtype among the muscarinic acetylcholine receptors, enhanced the abilities of cell migration and invasion in prostate cancer cells. The parasympathetic nervous system matters in cancer metastasis through the muscarinic acetylcholine receptors. Moreover, more parasympathetic nervous system functions and even the neuroendocrine system await exploration.

Up to this day, Atg5 was found to be tightly associated with the procedure of autophagy, including autophagosome extension and flexion, late endosome regeneration as well as its conjugation with Atg12, which is strongly required [34]. Besides, some scholars have discovered that the expression level of Atg5 is higher in benign prostatic carcinoma tissues than in benign prostate hyperplasia tissues. In this study, we observed an extremely suppressed expression of Atg5 after knocking down CHRM1 in PC-3 cells. Thereby, we determined whether the stimulation of CHRM1 acts to affect the levels of cell migration and invasion by Atg5. With a knockdown PCa cell model of Atg5, it was found that cell migration and invasion were significantly inhibited. On this basis, after adding CAR to activate CHRM1, it was found that the previous inhibitory effect was not significantly restored, suggesting that high expression of Atg5 could be a contributing factor in the early and malignant progression of malignant tumors.

AMPK*α*1 is activated with the process of stimulating Gq-coupled receptors. [35] Activating AMPK triggers autophagy in certain tissue [36]. Thus, we tentatively supposed that the Gq protein-linked CHRM1 might activate the AMPK-related pathway and induce autophagy to regulate tumor cell progression. Also, it is acknowledged that AMPK/mTOR pathway is fundamental in many procedures of nearly all kinds of tumors, including PCa. Moreover, AMPK/mTOR pathway was essential for the emergence and progress of autophagy [37–39]. Thus, in our study, the combined utilization of lentivirus transduction and CAR treatment revealed that, intriguingly, CHRM1 and Atg5 are positively related to AMPK/mTOR pathway in PCa cells. Even so, these findings await further investigation.

The certain limitation of this study lies in the lack of subsequent experiments in vivo and clinical verifications, which will be evaluated to ensure the accuracy of the results. To achieve this goal, further investigation needs to be done.

These studies reveal that regulating CHRM1-induced autophagy contributes to tumor development, including PCa. In this study, we started investigating the link between CHRM1 and autophagy activation in PCa, which has not been reported in previous studies to our knowledge. Therefore, we boldly speculate that it could be a potential therapeutic option for combining autophagy inhibitors and specific inhibitors of CHRM1 as a brand-new clinical medication in the prevention and treatment of advanced PCa.

## 5. Conclusion

In conclusion, the present findings indicated that autophagy induced by CHRM1 mediates migration and invasion targeting Atg5 via AMPK/mTOR pathway in prostate cancer cells. These findings uncovered that regulating muscarinic acetylcholine receptor M1, identified in this study, can be a promising solution for treating PCa.

## Figures and Tables

**Table 1:** Primer sequences used for qPCR.

Genes		Primer sequences (5′-3′)
CHRM1	Forward	CCGCTACTTCTCCGTGACTC
	Reverse	AACTGGATGTAGCACTGCCC

GAPDH	Forward	GCACAGTCAAGGCCGAGAAT
	Reverse	GCCTTCTCCATGGTGGTGAA

Atg12	Forward	CTAAGGAAGCCAGCTACAGG
	Reverse	TGGTGAATAATCCAGTTTGGGT

Atg16L	Forward	CTCTGGGATCTACGCAGCAA
	Reverse	CTCTGGGATCTACGCAGCAA

Atg5	Forward	CAACAGCTCCATCTCTCGAAC
	Reverse	CATGAAAGACTTACCGGACCA

BECN	Forward	TCAGAGATACCGACTTGTTCC
	Reverse	CTGTGTCTTCAATCTTGCCTT

Atg7	Forward	TGATAATGTCCTTCCCGTCAGC
	Reverse	ATGCAGCAATGTAAGACCAGT

LC3B	Forward	GCGTCTCCACACCAATCTCA
	Reverse	ACAATTTCATCCCGAACGTCT

## Data Availability

The data used to support the findings of the study can be obtained from the corresponding author upon request.

## Abbreviations

ATCC: American Type Culture Collection
Baf-A1: Bafilomycin A1
BECN1: BECLIN 1
CAR: Carbachol
CHRM1: Muscarinic acetylcholine M1 receptor
E64: Proteinase inhibitor E64
FN: Full nutrition
GPCRs: G-protein-coupled receptors
IHC: Immunohistochemistry
PCa: Prostate cancer
PEPS A: Pepstatin A
PIN: Pirenzepine
qPCR: Quantitative real-time PCR
SS: Serum starvation
TEM: Transmission electron microscopy
WB: Western blotting
3-MA: 3-Methyladenine.
